# Episiotomy

**Published:** 1885-09

**Authors:** 


### EPISIOTOMY.

Apropos of Dr. Morris' paper, "Incision of Sphincter Vaginæ in Certain Cases," which was published in our July number, and of which Dr. Bibb writes, in the present issue, we extract from a paper in the New York *Medical Journal* of August 15, by Dr. Reynold W. Wilcox, the following interesting facts.

"The operation of Episiotomy [nicking the parts, at the vaginal outlet, which are put upon the stretch during the second stage of labor—Ed.] does not seem to have received, at the hands of English and American writers on the subject of obstetrics, the attention to which its merits entitle it. In the majority of obstetrical treatises, the subject is dismissed in a few lines, or no allusion is made to it. If, on the contrary, it has attracted the attention of the author, it is often superficially discussed, or his erroneous preconceived ideas are apparent in his treatment of this subject. To one who has seen this operation as one of frequent, even-daily, occurrence in the lying-in wards of Vienna, this appears incomprehensible. It is the writer's intention to briefly point out its advantages and results, and, if possible, to deduce some impartial conclusions.

"Episiotomy is no new operation, nor is it an abandoned one recently resurrected, but one, although influenced by the fluctuations of obstetrical opinions, which has been in uninterrupted use for more than a century. If we read aright, the name was suggested by Ould, in his "Treatise on Midwifery," in 1742. (Parvin, in Trans. Am. Gyn. Soc., 1882, vol. vii., p. 151), although Michaels was the first to perform it in 1799."

Then follows a long and exhaustive paper on the subject, treating of laceration perinei and its causes, etc., which we have not space more than to alluded to.

